# AmpliconDuo: A Split-Sample Filtering Protocol for High-Throughput Amplicon Sequencing of Microbial Communities

**DOI:** 10.1371/journal.pone.0141590

**Published:** 2015-11-02

**Authors:** Anja Lange, Steffen Jost, Dominik Heider, Christina Bock, Bettina Budeus, Elmar Schilling, Axel Strittmatter, Jens Boenigk, Daniel Hoffmann

**Affiliations:** 1 Research Group Bioinformatics, Faculty of Biology, University of Duisburg-Essen, Essen, Germany; 2 Department of Biodiversity, Faculty of Biology, University of Duisburg-Essen, Essen, Germany; 3 Eurofins Genomics, Ebersberg, Germany; University of Milan-Bicocca, ITALY

## Abstract

High throughput sequencing (HTSeq) of small ribosomal subunit amplicons has the potential for a comprehensive characterization of microbial community compositions, down to rare species. However, the error-prone nature of the multi-step experimental process requires that the resulting raw sequences are subjected to quality control procedures. These procedures often involve an abundance cutoff for rare sequences or clustering of sequences, both of which limit genetic resolution. Here we propose a simple experimental protocol that retains the high genetic resolution granted by HTSeq methods while effectively removing many low abundance sequences that are likely due to PCR and sequencing errors. According to this protocol, we split samples and submit both halves to independent PCR and sequencing runs. The resulting sequence data is graphically and quantitatively characterized by the discordance between the two experimental branches, allowing for a quick identification of problematic samples. Further, we discard sequences that are not found in both branches (“AmpliconDuo filter”). We show that the majority of sequences removed in this way, mostly low abundance but also some higher abundance sequences, show features expected from random modifications of true sequences as introduced by PCR and sequencing errors. On the other hand, the filter retains many low abundance sequences observed in both branches and thus provides a more reliable census of the rare biosphere. We find that the AmpliconDuo filter increases biological resolution as it increases apparent community similarity between biologically similar communities, while it does not affect apparent community similarities between biologically dissimilar communities. The filter does not distort overall apparent community compositions. Finally, we quantitatively explain the effect of the AmpliconDuo filter by a simple mathematical model.

## Introduction

Amplicon-based environmental high-throughput sequencing (HTSeq) of markers such as SSU rRNA [1] have become a standard in biodiversity research. These methods have the potential to settle fundamental controversies about microbial diversity and distribution, including those resulting from the key problem of massive under-sampling of diversity, especially of the rare biosphere [2–5]. Consequently, large scale HTSeq projects have been initiated with high sample numbers and sequencing depths [6–8].

Modern HTSeq platforms, for instance Illumina MiSeq [9], deliver unprecedented amounts of clustered PCR fragments, leading to millions of single- or paired-end reads at moderate cost per run. Still, several problems remain, such as the relative short read lengths, or non-negligible error rates in PCR and sequencing steps. The latter potentially lead to overestimation and distortion of microbial biodiversity [10–12].

Two different kinds of errors are introduced during PCR amplification. First, polymerases used in PCR have error rates of about 1 substitution per 10^5^ to 10^6^ bases, depending on the type of polymerase [13]. Second, the PCR process can generate as a byproduct sequence chimeras by artificial recombination. Different studies on SSU data showed varying fractions of chimeric sequences [14, 15], from overall below 10% to more than 70% of sequence reads [11]. Moreover, the sequencing process itself introduces errors, dependent on the sequencing method [12, 16].

These errors and the methods chosen to eliminate them can have a strong impact on the biological interpretation of amplicon HTSeq data, and therefore great efforts have been invested in the development of best practice procedures for the analysis of amplicon HTSeq data [17–20]. Various strategies were devised to remove spurious sequences, such as (i) the removal of sequences that could not be taxonomically classified [21], (ii) discarding sequences with an abundance lower than a given threshold [22], and (iii) assignment of sequences by specialized clustering strategies [23]. Each of these strategies comes with its own drawbacks, for instance (i) true sequences might not have been taxonomically assigned so far, (ii) low abundance sequences might correspond to rare species, and (iii) sequence abundances are contaminated by reads from erroneous sequences and sequence resolution is decreased.

A basic assumption in the experimental sciences is that replicating an experiment under identical conditions increases the trustworthiness of re-occurring observations and reduces random noise. If we apply this assumption to a HTSeq experiment of a complex microbial community, we can state that a real sequence originating from organisms in the community should be observed in several replicates (except for extremely rare organisms). Moreover, we expect that an artificial sequence introduced by errors in the complex experimental HTSeq process should occur only in one replicate, if the error process is completely random and the error rate not too high. This reasoning suggests a minimal filter (“AmpliconDuo filter”) to eliminate artificial sequences: keep sequences that are observed in two replicates, discard sequences that are observed in only one replicate. In this way we are likely to keep real sequences (except those from extremely rare species), and to eliminate artificial sequences.

**Fig 1:**
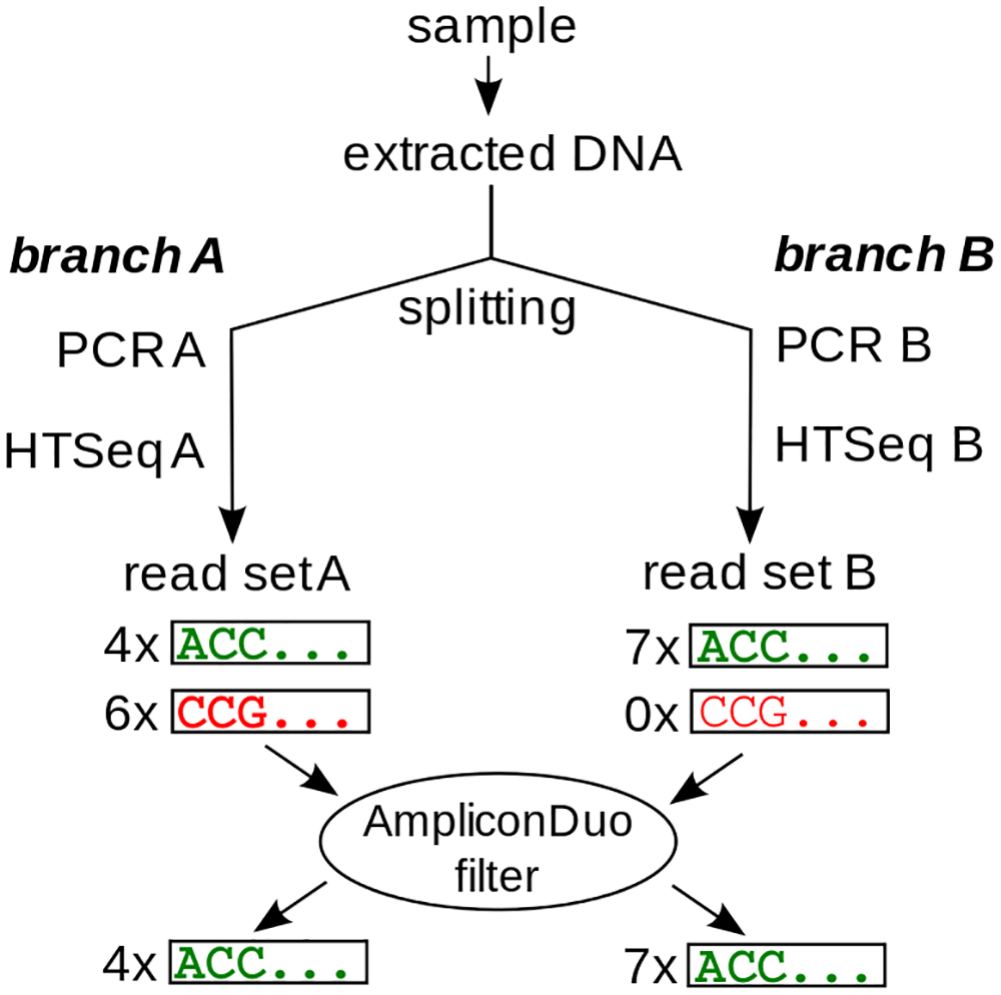
Principle of split sample approach with AmpliconDuo filter. DNA extracted from a sample is split into branches A and B. In each branch, an independent PCR and sequencing run is performed. Sequences occurring in both branches pass the AmpliconDuo filter (upper green sequence ACC… with 4 reads in A and 7 reads in B), while sequences occurring in only one branch are discarded (lower red sequence CCG…). Read numbers of both branches are retained for statistical analyses.

There are several important caveats that should be addressed in the above reasoning. First, sampling under identical conditions in the field is very difficult. Thus, to eliminate biological variability and focus on errors introduced by the technical process, we should take a single sample, extract DNA and then split this material into two halves, corresponding to two technical replicates. These two halves are then submitted independently to the same technical process of amplification by PCR and sequencing, so that we have two experimental “branches” for each biological sample that can be compared to identify real sequences and to discard artificial sequences, as we have argued above (Fig 1). Second, we have assumed that the errors are generated at random and not by a systematically biased error process. We will demonstrate that this assumption is likely to be correct for a fraction of errors, but that there are indications that it could be violated in the case of PCR chimeras [24].

As for the previously described filtering approaches, there are problems that potentially limit the scope of the split sample approach: First, the approach is either more expensive if sequencing depth is kept constant, or it permits not the same sequencing depth if cost is kept constant. As HTSeq becomes more affordable, this problem of cost/sequencing depth trade-off will become less relevant. Second, as mentioned before, ultra-rare species can occur in only one half of the split sample and are then eliminated. However, even if we acknowledge that the problem of ultra-rare species imposes a limitation, the increased sequence resolution achieved by omitting a pre-clustering step or stringent cutoff can still be valuable for studies of the fine structure of microbial biodiversity.

Protocols with several independent PCR branches have been used before (see e.g. Refs. [25, 26]), but these protocols usually have pooled material from different PCR branches to increase likelihood of true species recovery, and they did not exploit information from these branches as a means of filtering out spurious sequences. Recently, Esling *et al.* have published a study with mock communities in which they also demonstrate the benefits of combining different PCR branches [27].

In the following we present conceptual and computational tools for the implementation of the split sample approach that we have bundled in the R-package AmpliconDuo. Further, we apply the approach to several microbial samples, and we study the effect of the split sample approach on the apparent biodiversity and community composition. Since there is evidence that the underlying assumption of uncorrelated randomness may be violated for PCR chimeras, we also test the effectiveness of the approach for the removal of these artifacts by comparing outcomes with those of established methods for chimera detection.

## Materials and Methods

### Samples

Seven aquatic samples were analyzed for eukaryotic diversity: one from a small pond (“Uni pond”) at the University Campus in Essen, Germany, in July 2012, and six samples from Lake Fuschlsee (Austria) were taken fortnightly between June and September 2007. For the latter time series, we pooled for each time point three integrated samples covering the upper 10 *m* from the pelagic zone. Subsamples of 100 *mL* were filtered onto 0.2 *μm* polycarbonate filters for high-throughput sequencing. Filters were air dried and frozen at −80°*C* until further processing. All aquatic samples were filtered onto 0.2 *μm* polycarbonate filters (Merck Millipore, Billerica, MA, USA) until the filters clogged. Genomic DNA from aquatic samples was extracted using a CTAB Protocol (adapted from Ref. [28]). Also for analysis of eukaryote diversity, a soil sample was taken in July 2011 at a marsh near Gronau, Germany. Genomic DNA from the soil sample was extracted using the Fast DNA Spin Kit for Soil (MP Biomedicals, Santa Ana, California, USA) as recommended by the manufacturer, but with the following changes in protocol: bead beating to break cells was carried out three times using the Fast Prep 24 (MP Biomedicals, Santa Ana, CA, USA), and washing step with SEWS-M was carried out twice. Samples were split immediately after DNA extraction, and the following steps (PCR, sequencing) were applied independently to both halves of each sample.

Sampling the marsh was authorized by the Biologische Station Zwillbrock, Germany. No specific permissions were required for Lake Fuschlsee as confirmed by the Global Taxonomy Initiative National Focal Point in Austria and the Bundesamt für Wasserwirtschaft (Scharfling, Austria). The field studies did not involve endangered or protected species.

To test our protocol with prokaryotic data, we used material from a study on the effect of silver on microbial communities [29]. Shortly, the corresponding sample was taken from the same University pond in June 2013. Four subsamples of this sample were generated and subjected to treatment with silver nano-particles (here named “Pro4”), silver nitrate (“Pro3”), and no silver (“Pro1”, “Pro2”). DNA was extracted using the my-Budget DNA Mini Kit (Bio-budget Technologies GmbH, Krefeld, Germany) according to the manufacturers instructions but with an additional step, bead beating to break cells was carried out three times for 30 *s* using the Fast Prep 24 (MP Biomedicals, Santa Ana, CA, USA).

### PCR

**Fig 2:**
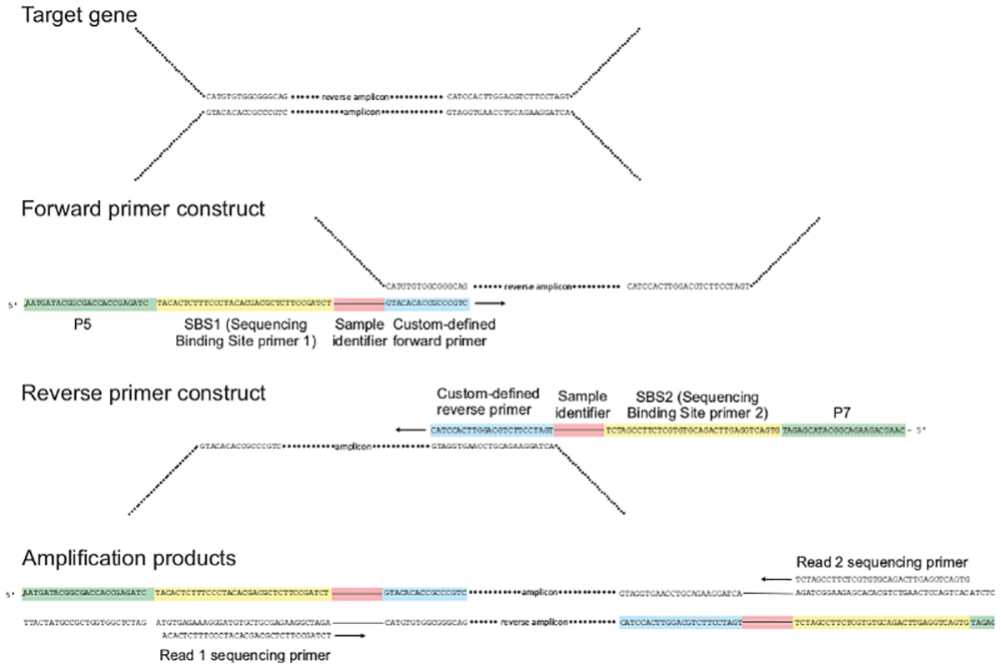
Primer construct and amplification products. The primers are composed of sequences specific to the sequencing platform (green), i.e. the P5 adaptor and the Illumina primer 1 for the forward primer and the P7 adaptor and the Illumina primer 2 for the reverse primer. Downstream follows a sample identifier starting with a poly-N (red) region and the custom defined primer (blue). In the reverse primer construct, the sample identifier was replaced by a poly-N region.

PCR was carried out with the Phusion high fidelity DNA Polymerase (Thermo Scientific) with 35 cycles and an annealing temperature of 71°*C*. Samples were amplified using primers consisting of Illumina-specific adapters, a sample identifier starting with a general poly-N region (S1 Table), and a user-defined primer (Fig 2). The applied amplicon strategy, based on user-defined primers and sample identifiers was adapted to the Illumina MiSeq platform. In case of the eukaryotic samples the forward primer Euk1391F (5’-GTA CAC ACC GCC CGT C-3’) and the eukarya-specific reverse primer Medlin B (5’-TGA TCC TTC TGC AGG TTC ACC TAC-3’) [30] were used to amplify the V9 region. For the prokaryotic data the SSU V2–V3 region was amplified with the bacteria specific primers 104F (5’-GGC GVA CGG GTG MGT AA-3’) and 515R (5’-TTA CCG CGG CKG CTG GCA C-3’).

### Sequencing

Sequencing of the different samples was carried out on a Illumina MiSeq platform at Eurofins Genomics. Prokaryotic samples were sequenced in paired-end mode giving rise to 2 × 300 nucleotides, whereas the eukaryotic samples gave raw reads with a length of 151 nucleotides.

### Quality filtering

For the eukaryotic diversity analysis, low quality tails were first removed from the reads. We discarded reads trimmed in this way that had an average Phred quality score [31] of less than 25. Additionally, all reads with at least one base with a Phred quality score of less than 15 were removed. Multiplex Identifiers (MIDs, S1 Table) were used to separate the different samples. Sequences with a mismatch in the primer or MID were discarded as well.

Samples for the analysis of prokaryotic diversity (S2 Table) were demultiplexed by Eurofins and required a perfect match in the MID sequence. The paired-end reads were assembled and quality filtered using PANDASeq version 2.7 [32]. Reads with uncalled bases, an assembly quality score below 0.8, a read overlap below 10, or a base with a recalculated Phread-score below 5 were discarded.

### Sequence clustering

The paired-end sequencing data were de-replicated with an R-script. For the single-end sequencing data, sequences with less than 115 nucleotides after trimming were discarded. Then, all remaining single-end reads were de-replicated with the same R-script. Sequence lengths in single-end data differed only in very few cases (< 0.025% of sequences in less than half of the samples with typically less than 20 reads). In those cases, shorter sequences were clustered with the matching full-length sequence with the highest abundance. Chimeras were identified using UCHIME [33] with its default settings either in *de novo* or reference mode. For the reference mode the Silva SSU Ref NR database release 119 was used as reference [34].

### Levenshtein distance

The generalized Levenshtein edit distance between sequences was calculated using the R function agrep in the R base package [35]. All editing costs were set to one, and the maximally allowed distance was increased from 1 to 4 in steps of 1.

### Taxonomy assessment

For all clustered reads, we used BLAST 2.2.30+ [36] and the Silva SSU Ref database release 119 to obtain taxonomic information [34].

### Statistical analyses

Significantly deviating read numbers between the two experimental branches (PCR and sequencing of each branch of a split sample) were detected with Fisher’s exact test applied to 2 × 2 contingency tables for all sequences *i*:

$$\left(\begin{array}{cc} r_{iAS} & r_{iBS}\\ \sum_{j\neq i}r_{jAS} & \sum_{j\neq i}r_{jBS} \end{array}\right)$$ (1)

with read number *r*_*iAS*_ of sequence *i* in experimental branch A of sample *S*, and the analogous for branch B. The false discovery rates *q*_*iS*_ for all sequences *i* in a sample *S* were computed from the *p* values of all sequences present in that sample using the method of Benjamini and Hochberg [37].

Measures of the discordance between branches A and B for the same sample *S* are $\Delta_{S\theta }^{r}$ (“read-weighted discordance”) and $\Delta_{S\theta }^{u}$ (“unweighted discordance”), the fractions of reads and sequences, respectively, with false discovery rate *q* below a chosen threshold *θ*, e.g. *θ* = 0.05:

$$\begin{array}{ccc} \Delta_{S\theta }^{r} & = & \frac{\sum_{i=1}^{n_{S}}\left(r_{iAS}+r_{iBS}\right)\delta \left(q_{iS}<\theta \right)}{\sum_{i=1}^{n_{S}}\left(r_{iAS}+r_{iBS}\right)} \end{array}$$ (2)

$$\begin{array}{ccc} \Delta_{S\theta }^{u} & = & \frac{\sum_{i=1}^{n_{S}}\delta \left(q_{iS}<\theta \right)}{n_{S}}, \end{array}$$ (3)

$$\begin{array}{ccc} \text{with}\;\delta (q_{iS}<\theta ) & = & \{\begin{array}{c} 1\;\text{for}\;q_{iS}<\theta \\ 0\;\text{for}\;q_{iS}\geq \theta \end{array} \end{array}$$ (4)

for number *n*_*S*_ of sequences detected in sample *S*. $\Delta_{S\theta }^{r}$ and $\Delta_{S\theta }^{u}$ lie between 0 (no discordance, i.e. no statistically significant deviations between experimental branches) and 1 (complete discordance). $\Delta_{S\theta }^{u}=0$ means that both branches A, B of the split sample yield the same set of sequences, $\Delta_{S\theta }^{r}=0$ means that, additionally, for each of the sequences read numbers in A and B are the same within an error margin determined by the chosen false discovery rate.

For the read-weighted discordance $\Delta_{S\theta }^{r}>0$ the deviation of read numbers of a sequence between the two branches is weighted with the average read number of that sequence in both branches: the more abundant a sequence, the more do significant deviations of reads of this sequence between A and B contribute to the read-weighted discordance $\Delta_{S\theta }^{r}$.

**Fig 3:**
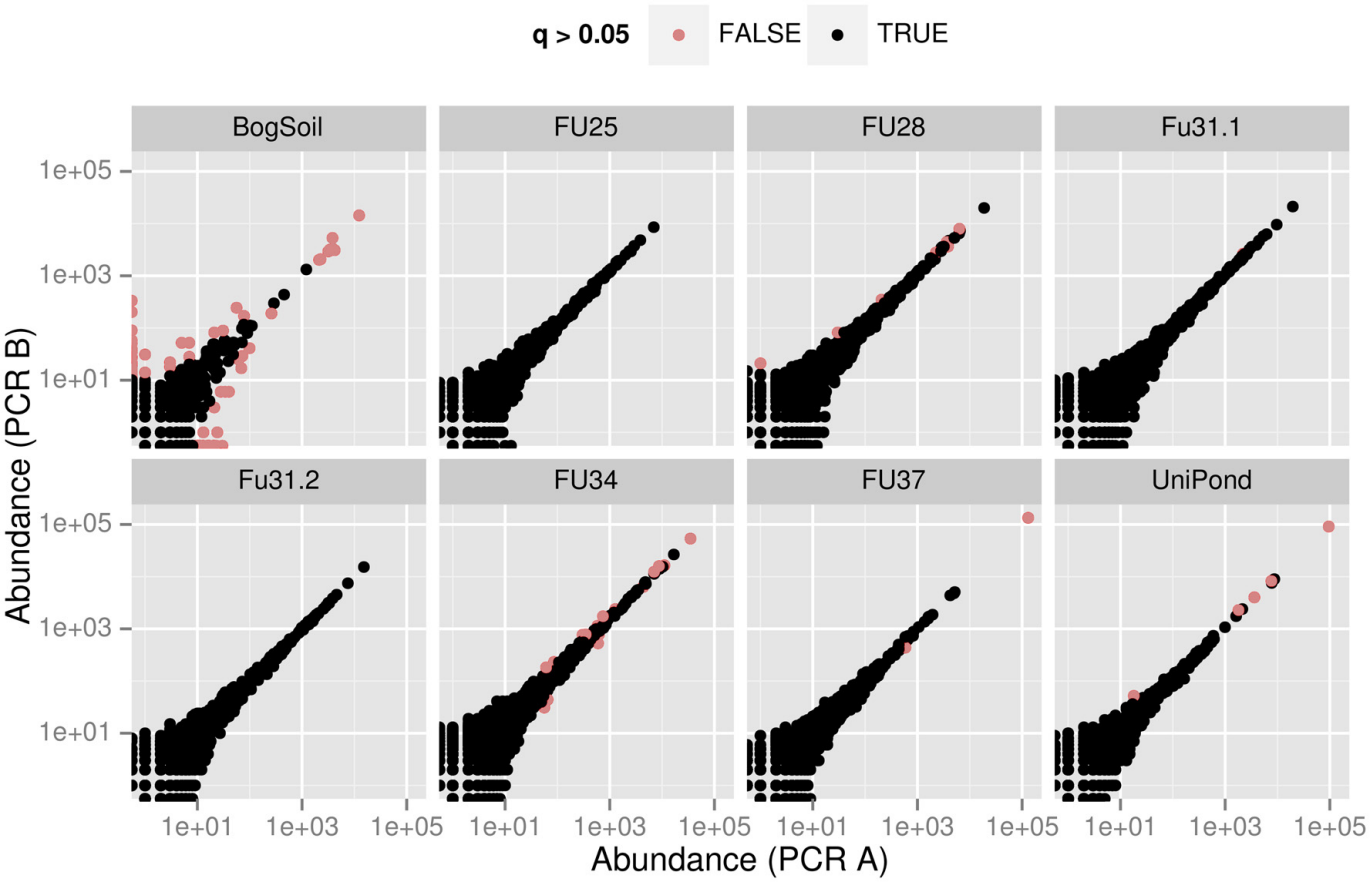
Discordance plot showing significant deviations of eukaryote read numbers between split samples. For each of the samples *S* an individual panel shows the logarithmically scaled pairs of read numbers (*r*_*iAS*_, *r*_*iBS*_) of unique sequences *i* in PCR branches *X* ∈ {*A*, *B*}. Red and black points correspond to, respectively, sequences with and without significantly deviating *r*_*iAS*_, *r*_*iBS*_ (false discovery rate *q* ≤ 0.05 or *q* > 0.05, respectively).

In each of the panels of the discordance plots Fig 3 and S1 Fig, $\Delta_{S,0.05}^{u}$ is the fraction of red points, and $\Delta_{S,0.05}^{r}$ is the fraction of sequence reads belonging to these points. Note that these fractions depend on the chosen false discovery rate. A strict false discovery rate of 0.05 (as used here) will generate higher Δ values than a more relaxed false discovery rate of e.g. 0.1 or 0.2.

In an ideal split sample, all points are black, meaning that for each sequence the fraction of reads of this sequence is the same in both experimental branches, within a confidence interval given by the false discovery rate. The higher the fraction of red points, the less reliable are community compositions or OTU abundances inferred from the respective sample.

Methods for the preparation of such figures and for the computation of the discordance values Δ^*r*^, Δ^*u*^ are implemented in the R-package AmpliconDuo that is freely available as platform independent source code from http://cran.r-project.org/web/packages/AmpliconDuo/.

The sets of *p*_*art*_ values at the end of the results section on “Discordance of split samples” were statistically evaluated for significant differences with the Brunner-Munzel test implemented in the R-package lawstat [38], and the effect size Cohen’s *d* as implemented in R-package effsize [39]. Given ranges for means and effect sizes of *p*_*art*_ are 95% confidence intervals assuming normal distributions.

### Community comparisons

For comparison of diversities between eukaryotic samples, sequence abundance vectors **r**_*iXS*_ were set up, one for each combination of one of the sampling sites *S* and experimental branch *X* ∈ {*A*, *B*}. Sequences assigned to Bacteria, Metazoa or Embryophyta were discarded. The vectors were submitted to agglomerative clustering using the complete linkage algorithm in R-function hclust. The distance measure used in the clustering procedure was the Jaccard distance measure *d*_*kl*_ of dissimilarity with values between 0 (samples *k*, *l* equal) and 1 (samples *k*, *l* dissimilar):

$$\begin{array}{ccc} d_{kl} & = & \frac{2}{1+b_{kl}}\;\text{with}\;b_{kl}=\frac{\sum_{i=1}^{N}r_{ik}+r_{il}}{\sum_{i=1}^{N}\left|r_{ik}-r_{il}\right|}, \end{array}$$ (5)

as implemented in the R-package vegan [40]. Indices *k*, *l* here denote combinations of experimental branch A, B and sampling site *S*. The Jaccard distance is a widely used measure of community similarity in ecology. It has several advantages over other measures, notably that it fulfills the triangle inequality [41]. Thus, it can be interpreted intuitively like a distance.

For an overview of taxonomic composition in all eukaryotic samples, the following taxa were grouped: Telonema (genus), Chrysophyceae, Diatomea (class), Kathablepharidae, Cryptomonadales, Ciliophora, Dinoflagellata, Cercozoa, Euryarchaeota, Prymnesiophyceae, LKM11, Choanomonada, Chytridiomycota (phylum), Dikarya, Chlorophyta (subkingdom), Cryptophyceae.rest, Alveolata.rest, Stramenopiles.rest (kingdom) and other/unassigned. Sequences with an alignment length below 95 (80%) and an identity below 93 were assigned to the group other/unassigned. Sequences assigned to Bacteria, Metazoa or Embryophyta were discarded.

## Results and Discussion

SSU amplicons were generated for eukaryotes (8 samples) and prokaryotes (4 samples). Each sample was equally split into two branches, A and B, and processed as described in Materials and Methods. In total we obtained 2577226 eukaryotic high-quality reads of 119 nucleotides, and 1332240 prokaryotic reads of lengths between 253 and 518 nucleotides. As eukaryote and prokaryote data showed the same overall patterns in the analyses, we have shifted most figures presenting prokaryotic data to the Supplemental Information.

### Discordance between split samples

We first determined for each split sample *S* the discordance values $\Delta_{S,0.05}^{r}$ and $\Delta_{S,0.05}^{u}$ between branches A and B (Tables 1 and 2) to assess the overall consistency between the branches. The unweighted discordance Δ^*u*^ was low in all cases, indicating a good consistency between the sequence sets in A and B branches. Conversely, the read-weighted discordance values Δ^*r*^ were much higher, reaching up to 83% (bog soil sample). This means that while there is little variation between the sets of sequences in A and B, the apparent sequence abundances reflected by the read numbers vary much more strongly between A and B.

**Table 1:** Discordance measures for eukaryotic samples.

	**Sample *S***	**$\Delta_{S,0.05}^{r}$**	**$\Delta_{S,0.05}^{u}$**
1	Bog Soil	0.832	3.75 × 10^−2^
2	FU25	0.000	0.00
3	FU28	0.104	4.18 × 10^−4^
4	FU31.2	0.000	0.00
5	FU31.1	0.014	5.60 × 10^−5^
6	FU34	0.285	6.55 × 10^−4^
7	FU37	0.658	2.01 × 10^−4^
8	Uni Pond	0.684	6.44 × 10^−4^

Discordance measures Δ^*r*^ (read-weighted), Eq (2)) and Δ^*u*^ (unweighted), Eq (3)) for eukaryotic samples. For all samples *S* the same false discovery rate threshold of 0.05 was used to define discordance.

**Table 2:** Discordance measures for prokaryotic samples.

	**Sample *S***	**$\Delta_{S,0.05}^{r}$**	**$\Delta_{S,0.05}^{u}$**
1	Pro1	0.235	3.76 × 10^−4^
2	Pro2	0.276	8.53 × 10^−4^
3	Pro3	0.591	3.93 × 10^−3^
4	Pro4	0.314	1.29 × 10^−3^

Discordance measures Δ^*r*^ (read-weighted), Eq (2)) and Δ^*u*^ (unweighted), Eq (3)) for prokaryotic samples. For all samples *S* the same false discovery rate threshold of 0.05 was used to define discordance.

The discordance plots Fig 3 and S1 Fig illustrate this finding in detail. $\Delta_{S\theta }^{u}$ in these figures is the fraction of red points, and $\Delta_{S\theta }^{r}$ is the read-weighted fraction of red points (red points in upper left and right contribute more). It becomes immediately clear that two of the samples, the eukaryotic bog soil sample and the prokaryotic Pro3 sample have a stronger discordance, possibly due to problems in sample processing. Note that e.g. in the case of Pro3 there are many sequences that are by about an order of magnitude more frequent in the B branch than in the A branch (upper red spike), while others have about the same abundance (black spike along the diagonal), and still others are somewhat less frequent in B (lower red rim of black spike). It is clear that quantitative community compositions inferred from such discordant samples are highly questionable. Thus, inspection of discordance quantities Δ^*u*^, Δ^*r*^ and discordance plots such as Fig 3 and S1 Fig available in the split sample approach help to identify problematic samples, and to avoid wrong interpretations of HTSeq microbial community data based on such problematic samples.

A note on why the discordance plots Fig 3 and S1 Fig are scaled logarithmically and which consequences this has for their interpretation: Visually, the log-scaling gives more room to the many low abundance sequences, while high abundance sequences are squeezed into a smaller area. This scaling mirrors the typical power-law distribution of reads in microbial communities: there are many low read count sequences and much fewer high read count sequences.

The log-scaling has a consequence for the appearance of deviations of read counts between PCR branches A, B. At the rare end in the lower left of the plot, even small absolute deviations show up as large distances from the diagonal, while at the high read count end in the upper right much larger absolute deviations that might be significant (red points) at the chosen false discovery rate shrink to only small apparent distances from the diagonal.

### AmpliconDuo filter

High-throughput sequencing brings us closer to discovering the complete composition of microbial communities, including the rare microbial biosphere. However, with the technology as of today, many sequences that could be interpreted as rare OTUs are actually sequences with errors introduced in PCR or sequencing. In the following we test a simple filtering strategy (“AmpliconDuo filter”) to eliminate such artifacts: we accept only sequences that occur in both branches A and B of a split sample.

The sequences removed by the AmpliconDuo filter will contain many true negatives, i.e. spurious sequences, induced by the introduction of random errors in the whole sequencing process. Sequences passing the filter have therefore a higher probability of being true positives, i.e. real sequences. On the other hand, the approach will suffer from false negatives as we miss extremely rare real sequences that are sampled in only one branch of the split sample. At some point, we have to accept this particular error as a natural consequence of the observation method: we cannot observe arbitrarily rare sequences in a limited sample volume. It is beyond the scope of this work to clarify how close we have come to this natural boundary. Instead, we will in the following sections study the effect of the AmpliconDuo filter by analyzing properties of removed and retained sequences.

**Fig 4:**
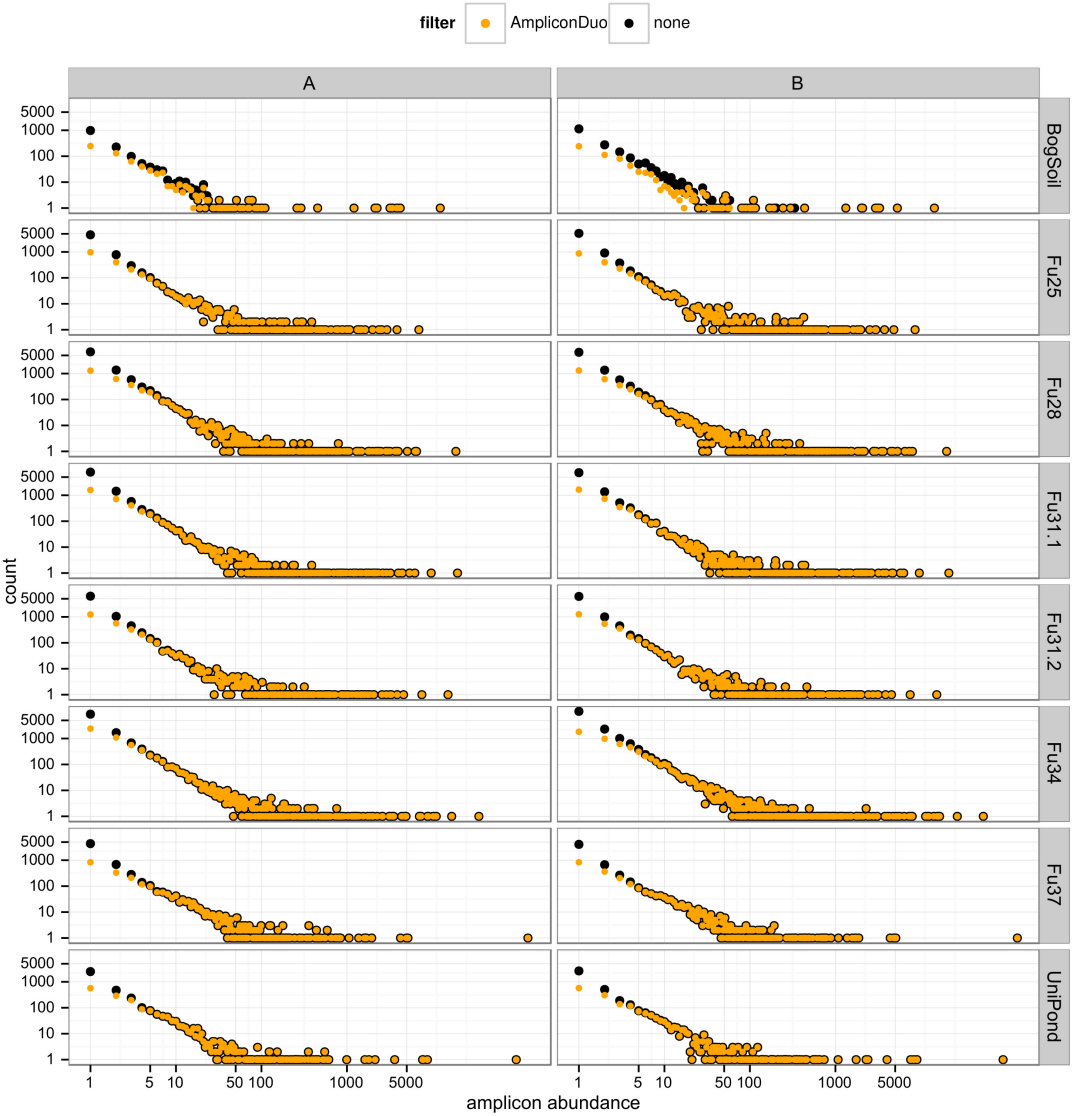
Effect of AmpliconDuo filter on spectrum of read numbers for eukaryotic data. Columns A and B are experimental branches of the split sample, rows correspond to sampling sites. Number of sequences before and after AmpliconDuo filtering are plotted as black and orange dots, respectively. Both axes have logarithmic scales.

All samples have approximately the same power law behavior of sequence abundance as function of read number (Fig 4 and S2 Fig) showing up as a linear relationship in the log-log plots of sequence counts vs. sequence abundance (or number of reads). By far most of the sequences removed by the AmpliconDuo filter occur with low read numbers, especially singletons: 72–84% of eukaryotic singletons and 87–94% of prokaryotic singletons are removed as they are observed in only one experimental branch of the split samples.

Beyond about ten reads (see also quantitative theoretical argument in section “Is AmpliconDuo filter effectively removing chimeras?”) the number of sequences is almost unaffected by the AmpliconDuo filter, except in those cases that have conspicuous discordance (Fig 3 and S1 Fig, Tables 1 and 2). Namely in the eukaryotic bog soil sample and the prokaryotic Pro3 and Pro4 samples there are some apparent sequences with higher abundances that occur only in one branch A or B and that are therefore discarded by the AmpliconDuo filter; in Fig 4 and S2 Fig these cases are visible as black dots at higher abundance that are not covered by orange dots. For non-problematic samples, the AmpliconDuo filter has a perceivable impact only for the measurement of the rare microbial biosphere. However, even after filtering, this rare end of the abundance spectrum contributes the highest numbers of distinct sequences because the AmpliconDuo filter accepts a considerable fraction of low abundance reads.

We assume that many of the spurious sequences occurring in only one experimental branch of the split sample are the results of artificial random point mutations introduced into real sequences by errors in PCR or sequencing. If this assumption is true, many of the sequences removed by the AmpliconDuo filter should be closely related to real sequences that pass the filter. We have tested this with our data (S3 and S4 Figs) by counting the number of removed sequences with Levenshtein distances of one to four editing operations (single nucleotide mutations, deletions or insertions) to retained sequences. With a Levenshtein distance *L* = 1 we recovered 47–71% of discarded eukaryote and 23–43% of discarded prokaryote sequences. Allowing an editing distance of up to *L* = 4 recovered 64–91% and 40–62% of eukaryote and prokaryote sequences, respectively, removed by AmpliconDuo filtering.

The pattern of fractions of filtered-out sequences explained by increasing *L* to sequences passing the AmpliconDuo filter is remarkably consistent over all samples, including both eukaryotes and prokaryotes: Most bars in S3 Fig are dominated by *L* = 1, followed by decreasing contributions from *L* = 2 to *L* = 4. This is a pattern that could arise from two plausible causes: (1) If the experimental procedure (mainly PCR and sequencing) introduces single random mutations in a Poisson-like process with a low rate, we expect the greatest contribution from *L* = 1 (one artificial mutation), followed by *L* = 2 (two artificial mutations), etc. (2) The grading of frequencies from *L* = 1 to higher *L* could reflect the real distribution of genetic changes in SSU sequences in microbial populations.

If the first of these two plausible causes is true, we expect a certain pattern of artificial mutants: each real sequence will be accompanied by a number of artificial mutants that differ from the original real sequences by one mutation or a few mutations, i.e. a kind of “halo” of artificial mutants around the real sequence. The density and size of this halo will depend not so much on the origin of the sample or the type of organism, but mainly on the features of the experimental protocol, especially PCR and sequencing. Since we have only two variants of the protocol, namely the single-read sequencing in the eukaryote samples and paired-end sequencing in the prokaryote samples (see Methods), we expect only two types of halos of artificial random mutants.

If the probability of an artificial mutation per nucleotide *p*_*art*_ is small, most artificially mutated sequences will differ by one artificial mutation from real sequences. (Note that *p*_*art*_ quantifies the artificial mutation rate per nucleotide that remains *after* quality filtering of sequences (see Methods); the corresponding error rate in the raw sequences *before* quality filtering is likely to be much higher). Under this assumption we anticipate:

$$\begin{array}{cc} n_{L=1,rej} & \approx p_{art}\sum_{i=1}^{n_{acc}}\ell_{i}r_{i},\;\text{or}\; \end{array}$$ (6)

$$\begin{array}{cc} p_{art} & \approx \frac{n_{L=1,rej}}{\sum_{i=1}^{n_{acc}}\ell_{i}r_{i}} \end{array}$$ (7)

with the number *n*_*L* = 1, *rej*_ of sequences rejected by the AmpliconDuo filter that have a Levenshtein distance *L* = 1 to an accepted sequence, the sequence lengths ℓ_*i*_, and the numbers *r*_*i*_ of reads of the *n*_*acc*_ accepted sequences. Eq (6) expresses that the rejected sequences with *L* = 1 to accepted sequences are dominated by artificial random point mutations, generated with a probability *p*_*art*_ per nucleotide. The longer a sequence is and the more reads it has, the more probable that an artificial mutation is introduced into that sequence.

**Fig 5:**
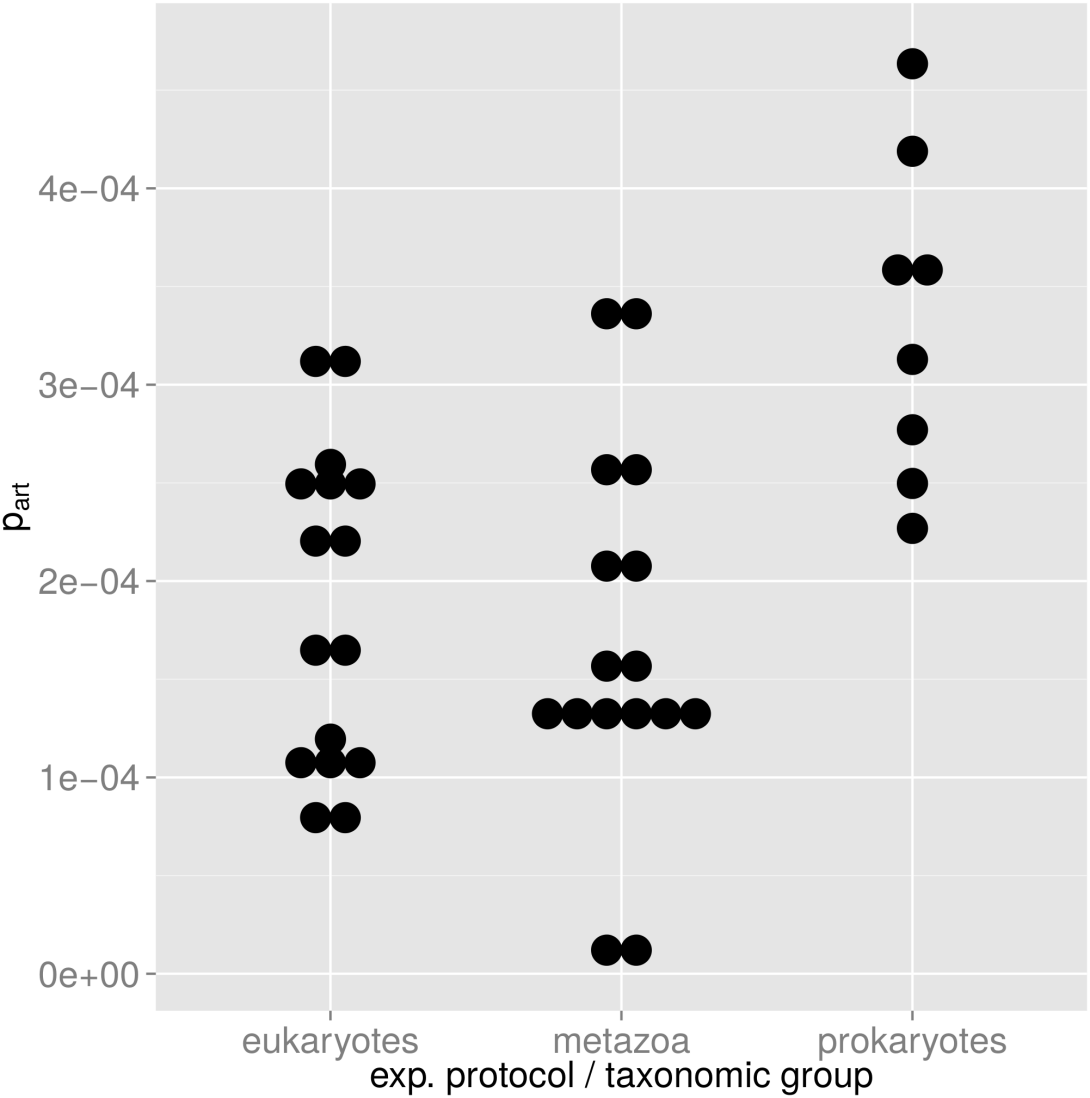
Distribution of probability *p*_*art*_ of artificial random mutations. Each dot corresponds to one *p*_*art*_ value computed for one experimental branch A or B according to Eq (6). In the plot, *p*_*art*_ values are binned in intervals of 1/30 of their total range. Eukaryotes and metazoans (first two columns) have both been analyzed with the same single-read protocol, and the mean *p*_*art*_ of these two groups are not significantly different. For the prokaryotic samples that have been analyzed with a paired-end protocol, we have a higher *p*_*art*_.

If our model is correct, *p*_*art*_ will mainly depend on the experimental protocol, i.e. it will be more or less the same for different samples as long as the same experimental protocol is used. Fig 5 supports this model: For each of the two protocols we can estimate a mean *p*_*art*_ with 95% confidence intervals that do not overlap: For the single-read protocol (eukaryotes) we have *p*_*art*_ = (1.9 ± 0.4) × 10^−4^. For the paired-end protocol (prokaryotes) we have *p*_*art*_ = (3.3 ± 0.7) × 10^−4^. A Brunner-Munzel test between the two *p*_*art*_ distributions yields a p-value of 5.7 × 10^−5^ for the null hypothesis of equal means of the two distributions. Cohen’s *d* is estimated at 1.79 ± 1.1, indicating a medium to large effect of the experimental protocol on *p*_*art*_.

The difference in *p*_*art*_ could also be due to the fact that we have applied each of the two different protocols to one specific taxonomic group, i.e. microbial eukaryotes vs. prokaryotes so that the difference in *p*_*art*_ could be a consequence of biology rather than of technology. To test this possibility, we have analyzed in the same way metazoan sequences that were obtained as bycatch in the eukaryote samples. It is reasonable to assume that the true genetic SSU diversity in this metazoan bycatch is much lower than the genetic SSU diversity in the microbial eukaryotes in these samples. This means that if *p*_*art*_ is mainly influenced by the true genetic diversity, we should see a trend to lower *p*_*art*_ values in metazoans. Conversely, if our hypothesis of a halo of artificial sequences described by *p*_*art*_ is correct, we should see the same value of *p*_*art*_ in the metazoans, irrespective of their lower genetic diversity since the experimental protocol for microbial eukaryotes and metazoans is the same. This outcome should not be influenced by a possible PCR bias introduced by the primers that had been optimized for microbial eukaryotes, since this bias should affect both experimental branches A and B in the same way. The middle column in Fig 5 shows that microbial eukaryotes and metazoans have about the same *p*_*art*_: for microbial eukaryotes *p*_*art*_ = (1.9 ± 0.4) × 10^−4^, and for metazoans *p*_*art*_ = (1.7 ± 0.5) × 10^−4^. A Brunner-Munzel test cannot reject the null hypothesis that the microbial eukaryote and metazoan *p*_*art*_ are the same (p-value 0.78).

These results are consistent with the model that at least the dominating group of sequences with *L* = 1 rejected by the AmpliconDuo filter can be explained as arising mainly from artificial random mutations introduced by the experimental process.

The extension of the rejection pattern to higher abundances, especially for the rather discordant bog soil samples (top row in S3 Fig) and the prokaryotic samples (S4 Fig) again highlights a property that distinguishes the AmpliconDuo filter from filter schemes that merely cut off low abundance reads. While AmpliconDuo filters out many low abundance sequences, it still retains many of these sequences and thus offers the possibility of a more reliable detection of rare OTUs. On the other hand, AmpliconDuo discards also higher abundance sequences that are not reliably occurring in the two branches of a sample and that thus may be PCR artifacts.

To conclude this section, we compare our results to independent results published recently by Esling *et al.* [27]. They applied intersections between split samples (“replicate intersections”) to several mock communities of known composition. If applied to samples that are split in two replicates, filtering by such replicate intersections is identical to the application of the AmpliconDuo filter. Esling *et al.* report for their mock communities, composed of mixtures of 4 to 40 known clones, that intersections of two replicates on average remove 87.4% of erroneous sequences, corresponding to 35.5% of the reads. In the experiments by Esling *et al.*, the total number of sequences in these mock community experiments is by far dominated by erroneous sequences, with real sequences making up at most about 10% of total sequences, typically much less. Hence, we can approximately equate the percentage of removed sequences with the percentage of total sequences, which we can directly compare with our results.

For our microbial community data, the AmpliconDuo filter removes in the eukaryote samples (single-end sequencing protocol) on average 62.1% (±4% sample standard deviation) of the total sequences and 4.3% (±1.9%) of reads (S3 Table). For the prokaryote samples, sequenced with a paired-end protocol as in Ref. [27], the AmpliconDuo filter removes 84.0% (±3.3%) of total sequences and 29.0 (±6.0%) of reads.

Thus, there is an overall qualitative agreement between the low complexity mock community data of Esling *et al.* [27] and our high complexity microbial community data as far as the disparity between fractions of removed sequences and reads are concerned: both mock and microbial community data show preferential removal of many low abundance sequences, leading to fractions of rejected sequences clearly above 50%, and lower fractions of rejected reads, clearly below 50%.

Moreover, the numbers above show good quantitative agreement between Esling *et al.* and our prokaryote data, both of which were generated with a paired-end sequencing protocol on the same Illumina MiSeq platform. Conversely, the quantitative agreement with our eukaryote single-end data is less good. This is consistent with our results for *p*_*art*_, which was on average significantly lower for the single-end protocol than for the paired-end protocol.

### Community comparisons

A filtering procedure should preferentially remove spurious sequences and thus emphasize true biological effects. One way of testing this is to observe how the filtering affects apparent similarities of microbial communities. Application of a filtering procedure that removes random errors in community sequence data should have two effects. First, since random errors diminish apparent similarity between communities, removal of random errors should generally increase similarities between communities. Second, this increase should grow with true similarity: for samples that are biologically close to identical (for instance the two halves of a split sample), random noise explains most of the apparent community difference and thus removal of random noise should increase apparent similarity, or, in terms of the Jaccard distance *d*_*kl*_ between communities *k* and *l* (Eq (5)): *d*_*kl*_ should drop to a small value. On the other hand, for samples that are truly dissimilar, removal of random noise will diminish *d*_*kl*_ only slightly.

**Fig 6:**
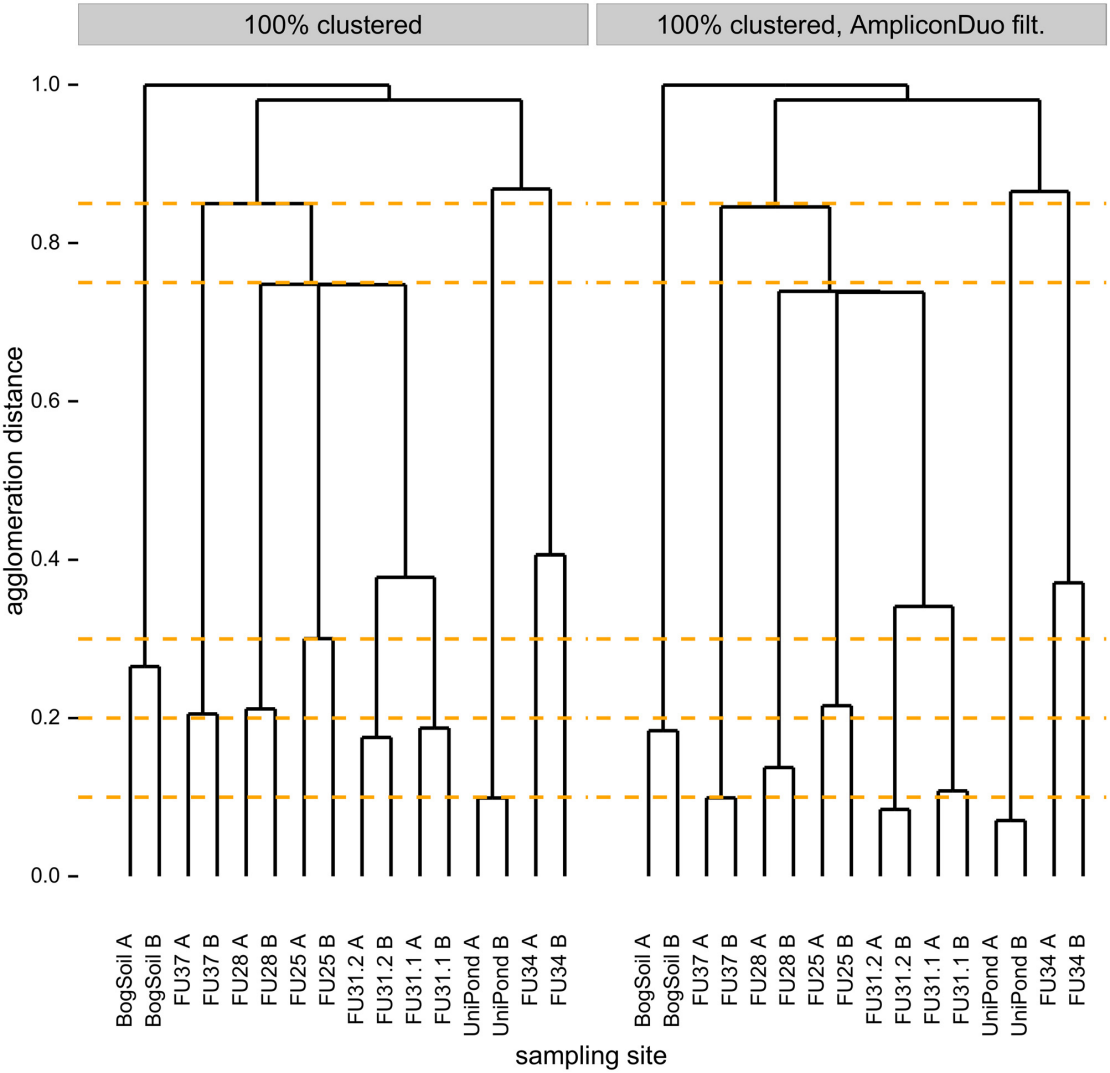
Effect of AmpliconDuo filtering on apparent eukaryote community similarities. Comparison of samples with respect to Jaccard distances *d*_*kl*_, Eq (5), between sequence abundance vectors. Left panel: Sequences clustered at 100% identity. Right panel: Sequences clustered at 100% identity and excluding sequences observed in only one branch of a split sample (AmpliconDuo filter).

In fact, we observe that AmpliconDuo filtering, i.e. removal of sequences that occur only in one branch of a split sample, has the predicted effects on community (dis-)similarities as expressed by Jaccard distances (Fig 6). We compared community compositions based on unfiltered data across all eukaryotic samples (see Methods section on community comparisons), including the two branches of each split sample that should be close to identical, and biologically different samples from different sites (left panel of Fig 6). Then we repeated all these comparisons after application of AmpliconDuo filtering (right panel of Fig 6). For clarity we emphasize that the AmpliconDuo filter was only applied to branches A and B of the same sample, not to combinations of branches of different samples. The comparison of the two dendrograms shows that AmpliconDuo filtering generally decreases Jaccard distances, i.e. it increases apparent similarities: all agglomeration distances in the dendrogram drop. Moreover, the strongest drops of Jaccard distances, or increases in similarity, occur between biologically most similar samples, namely between the A and B branches of split samples. For instance, the Jaccard distance between FU37A and FU37B drops from 0.2 to 0.1 as we apply the AmpliconDuo filter. For the biologically more distinct samples with their higher Jaccard distances, e.g. between sampling sites FU25, FU28, and FU31 where we have an agglomeration distance of about 0.75 before the filtering step and only slightly less afterwards (compare change of branch point relative to yellow dashed visual helper line). The larger drops in Jaccard distance between more similar samples, combined with smaller drops for less similar samples, emphasize true biological community differences.

Fig 6 also shows that AmpliconDuo filtering does not completely erase Jaccard distances between corresponding A and B branches. This cannot be expected since there remains a sampling error between both halves of a split sample. This sampling error causes many small read number variations between the sample halves that add up in Eq (5) to a finite Jaccard distance. The only way a Jaccard distance of zero can be obtained is by a denominator of zero in *b*_*kl*_ in Eq (5), leading to 1 + *b*_*kl*_ → ∞ in the denominator of *d*_*kl*_ and thus to *d*_*kl*_ → 0. The sampling error makes such a perfect fit between split samples very unlikely. Consequently, there is a trend to larger residual Jaccard distances for higher sequence richness, especially for rare sequences with their relatively high sampling errors (compare Fig 6 and S4 Table). For instance, FU34 has by far the highest sequence richness and also the highest residual Jaccard distance, while UniPond has the lowest sequence richness of all aquatic samples and the lowest residual Jaccard distance. For the problematic, high-discordance BogSoil sample this relation does not hold: it has relatively high residual Jaccard distance but low sequence richness.

In summary, AmpliconDuo filtering in general decreases Jaccard distances, and it does so the more the closer the biological relatedness. This increases the Jaccard distance gaps between biologically similar and dissimilar communities, and thus effectively increases biological resolution.

**Fig 7:**
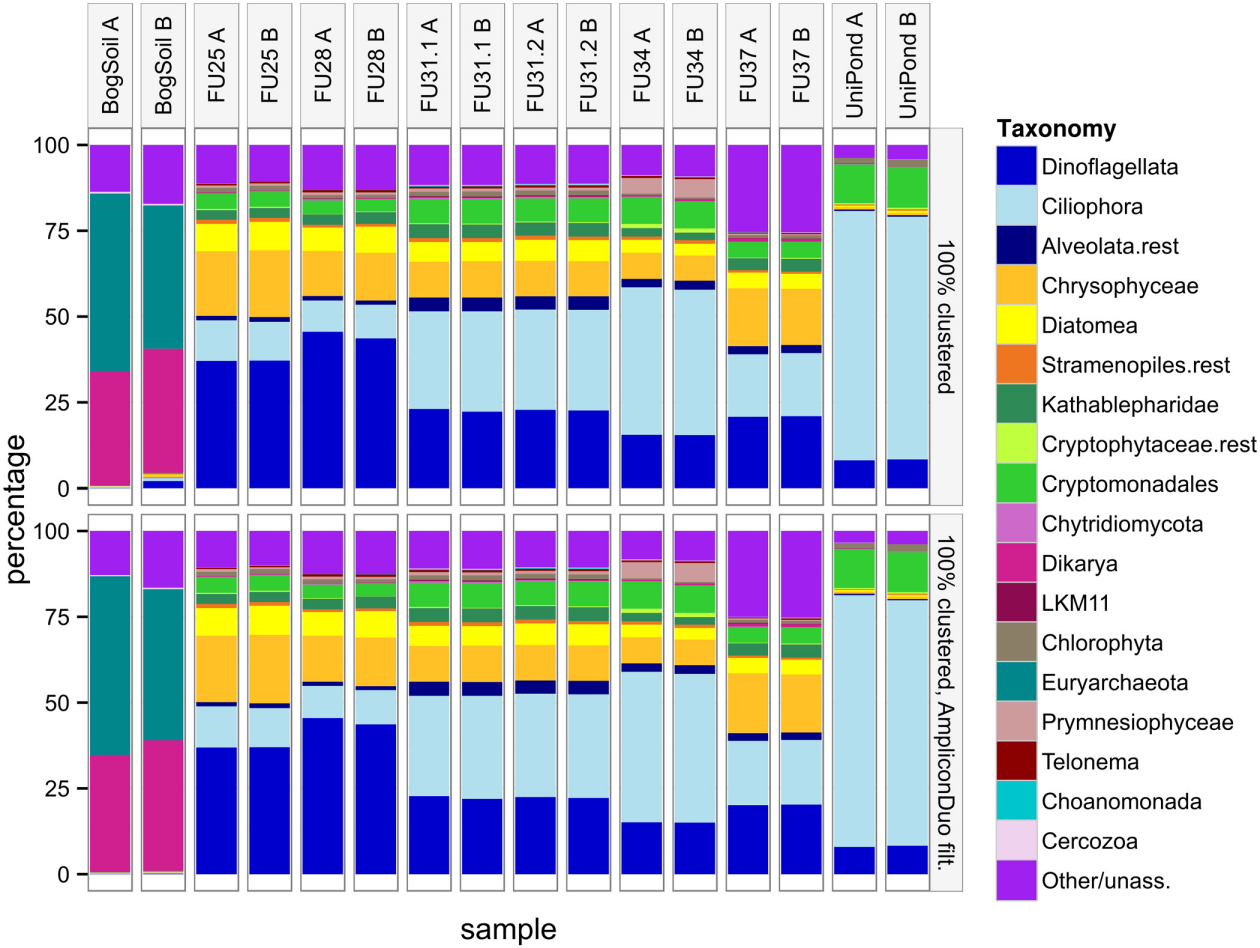
Taxonomic composition of eukaryotic communities before (top) and after (bottom) AmpliconDuo filtering. In the bog soil sample, many archaean taxa were captured by the broad eukaryotic primers used in this study. Archaea were therefore not discarded from the bog soil sample for this community comparison.

We checked whether the dendrograms in Fig 6 can be understood based on the fractions of taxonomic groups. Fig 7 shows that in general branches A and B of the same sample are more similar to each other than to samples of other sites. As in the dendrograms, the bog soil sample is the least similar sample to the remaining samples. The surprising agglomeration of FU34 with the Uni pond sample instead of the other samples from lake Fuschlsee becomes understandable if we consider that FU34 is extreme among the FU samples because it has the highest fractions of Ciliophora and Cryptomonadales, and the lowest fractions of Dinoflagellata, Chrysophyceae, and Diatomea. All these differences to the other FU samples bring FU34 closer to the composition of the Uni pond sample.

At the resolution of Fig 7, only a few changes between top and bottom panel are clearly visible, e.g. the loss of some of the rarer groups in bog soil branch B. Overall, comparison of the two panels suggests that application of the AmpliconDuo filter does not change community compositions. This is consistent with the conserved topology between the two dendrograms in Fig 6. Together with the discussion of S3 Fig where we saw that most of the sequences eliminated by the AmpliconDuo filter were slight, probably random variants of non-eliminated sequences, we conclude that these variants are spread homogeneously over all taxonomic groups.

### Is AmpliconDuo filter effectively removing chimeras?

As outlined in the introduction, formation of chimeras, i.e. artificial recombination of unrelated nucleotide sequences, is a well-known PCR artifact. If this process was completely stochastic with random breakpoints between randomly recombining sequences, we could hope for low numbers of chimeras that occur in both independent PCRs of the two branches of each split sample. Alternatively, chimera formation could be biased towards specific breakpoints and specifically recombining sequences [24], in which case we would expect recurrent chimeric sequences in the two branches. In the first scenario, the AmpliconDuo filter would effectively remove chimeras, in the alternative scenario, the filter would be not as effective against chimeras but let a fraction of them pass (false positives).

To test the suitability of the AmpliconDuo filter as means for chimera removal, we analyzed sequences before and after AmpliconDuo filtering with the established UCHIME method [33] for chimera identification.

For the eukaryotic samples studied here, the fraction of sequences recognized by UCHIME as chimeras was always below 1.5% with a maximum number of 242 reads, no matter whether we used UCHIME in *de novo* or in reference mode (data not shown). This small fraction could be explained by the short effective sequence lengths of 119 nucleotides, which might have limited the chance of observing artificial recombination or of confident recognition of chimeras by UCHIME.

Conversely, in the prokaryotic samples, with sequences almost three times as long as the eukaryotic ones, the fractions of chimeras were much higher. Depending on whether we used UCHIME in *de novo* or reference mode (see Methods), 11–23% or 23–52%, respectively, of the sequences were labeled as chimera. While UCHIME in reference mode identified much more low abundance chimeras, the agreement between the two UCHIME modes was overall high. The read numbers of chimeric sequences reached up to 799.

**Fig 8:**
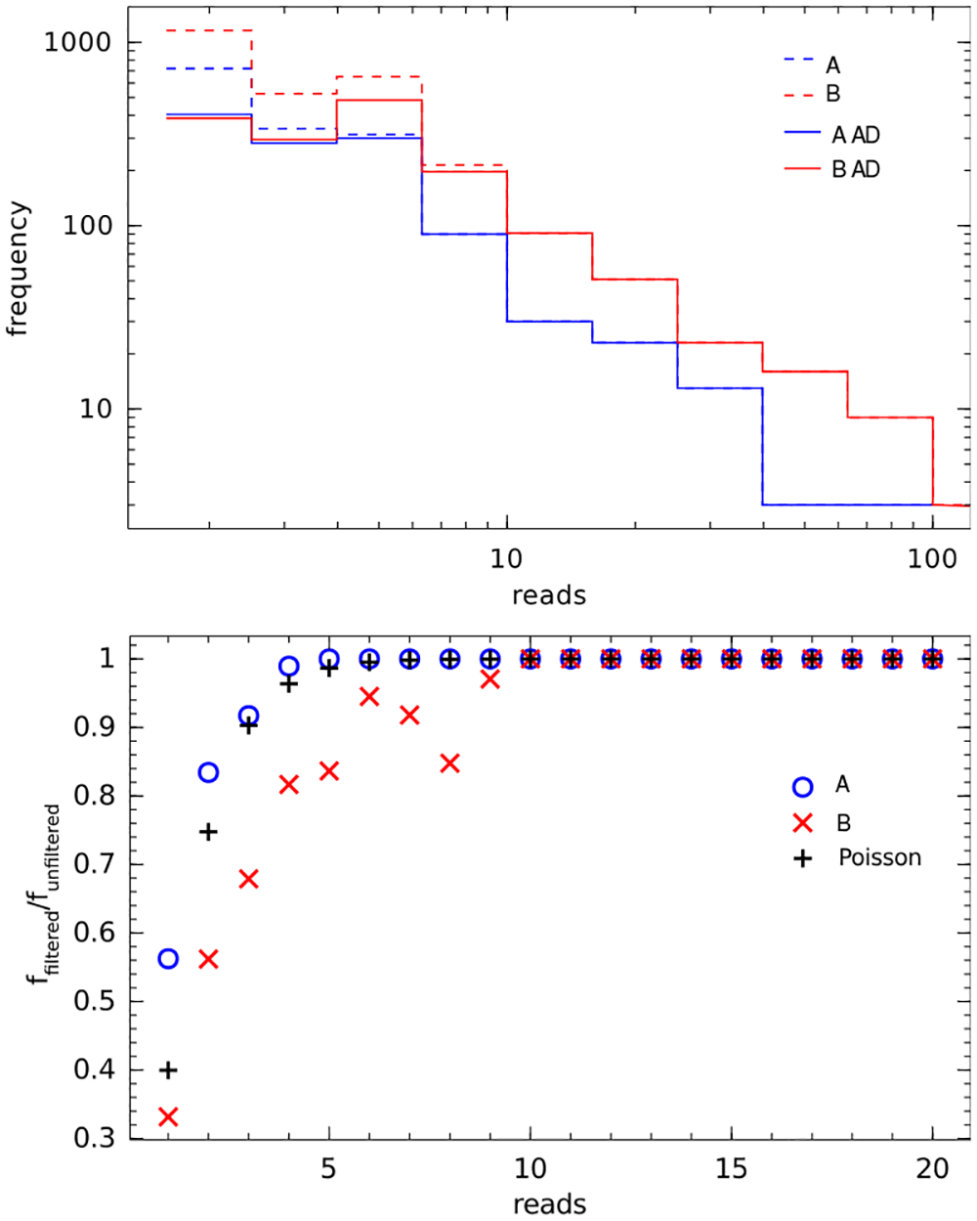
Effect of AmpliconDuo filtering on chimeras for prokaryotic sample Pro2. Chimeras defined by being recognized by UCHIME in *de novo* mode with score ≥ 1. Top: Frequency of chimeras in branches A, B of split sample as function of their read numbers, before (dashed lines) and after (solid lines) application of AmpliconDuo filter. Bottom: Fraction of chimeras passing the AmpliconDuo filter (*f*_*filtered*_/*f*_*unfiltered*_) for read numbers 1 to 20 in both branches A, B, and corresponding prediction *P*(*r*_*iA*_, *r*_*iB*_ ≥ 1) using the Poisson model in Eq (8) with *λ*_*i*_ = 1, 2, …, 20.

When we studied the effect of the AmpliconDuo filter on the prokaryotic chimeras, we found that the AmpliconDuo filter removed only low abundance chimeras, and even there the removal was incomplete. Generally, effectiveness of the AmpliconDuo filter for chimera removal decreased with increasing chimera abundance and was negligible for sequences with read numbers of ten or more. The top part of Fig 8 illustrates this for a prokaryotic sample (see S5 Fig for a detailed breakdown of samples Pro1 and Pro3).

This result indicates that the alternative scenario of biased chimera formation cannot be rejected, since this scenario explains the frequent recurrence of the same chimera in both branches of the split sample. With our experimental data we also cannot reject the hypothesis that a fraction of sequences labeled as chimeras correspond to real prokaryotes.

The decreasing filtering effect of AmpliconDuo on chimeras, ceasing altogether for chimeras with ten or more reads can be quantitatively explained as a consequence of random sampling, as will be shown in the following. In order to pass the AmpliconDuo filter, we have to observe a chimera sequence *i* in each of the two branches A, B of the split sample at least once, which means that for the corresponding read numbers of *i* in these branches we have to have *r*_*iA*_, *r*_*iB*_ ≥ 1. The probability of a sequence *i* occurring in one branch of the split sample at least once is one minus the probability of that sequence not occurring in that branch, i.e. *P*(*r*_*i*_ ≥ 1) = 1 − *P*(*r*_*i*_ = 0). The probability *P*(*r*_*iA*_, *r*_*iB*_ ≥ 1) of a sequence *i* occurring at least once in each of the two independent branches A, B is then *P*(*r*_*iA*_, *r*_*iB*_ ≥ 1) = *P*(*r*_*i*_ ≥ 1)^2^ = (1 − *P*(*r*_*i*_ = 0))^2^. If we assume that the probability of each chimera *i* is governed by a Poisson distribution with a specific *λ*_*i*_, the probability *P*(*r*_*iA*_, *r*_*iB*_ ≥ 1) becomes:

$$\begin{array}{c} P\left(r_{iA},r_{iB}\geq 1\right)={\left(1-\frac{\lambda_{i}^{0}}{0!}e^{-\lambda_{i}}\right)}^{2}={\left(1-e^{-\lambda_{i}}\right)}^{2}. \end{array}$$ (8)

This is the probability that a sequence *i* with read number expectation value *λ*_*i*_ is not filtered out by the AmpliconDuo filter. We can estimate this probability directly from our experimental data by dividing the number of chimeras passing the AmpliconDuo filter by the total number of chimeras (as labeled by UCHIME). The example of Pro2 shows that the agreement between theoretical prediction and experimental values is good (bottom panel of Fig 8): Both start at probabilities of a 0.3 to 0.5 for chimeras with expected read numbers of 1 and all converge to probability 1 at about read numbers of 10. The lower than expected values for experimental branch B in that figure are in agreement with the non-negligible discordance of sample Pro2 (Table 2), and the clearly manifest red rim in the Pro2 panel of the discordance plot S1 Fig below the black diagonal spike.

Note that the Poisson argument also explains why in Fig 4 and S2 Fig raw data and data filtered with AmpliconDuo consistently converge at around read numbers of five to ten. Due to the sampling error, we will lose a fraction of rare real sequences that by chance are sampled in only one of the two branches. This sampling error can be neglected for rare sequences with more than about five to ten reads according to this Poisson argument. On the other hand, the AmpliconDuo filter will remove many artificial sequences irrespective of abundance, as discussed above.

### Conclusions

In this study we have developed methods for the characterization of HTSeq data from microbial communities, using a split sample protocol. This included, firstly, numerical and graphical means for characterizing discordance between the two branches of the split sample, as implemented in the R-package AmpliconDuo, and secondly, the application of the “AmpliconDuo filter”, a simple filter protocol for the removal of sequences with random errors. Both are generally applicable to HTSeq amplicon data and neither restricted to microbes nor to the SSU gene and Illumina MiSeq data. They are equally suitable for the analysis of HTSeq data from other diverse genetic systems, e.g. RNA viruses in a patient, or antibody or T-cell receptor genes.

The numerical and graphical characterization allowed a rapid identification of problematic samples, such as the eukaryotic bog soil sample (Fig 3) and the prokaryotic Pro3 sample (S1 Fig).

The AmpliconDuo filter protocol increased the biological resolution in the sense that the similarity of biologically more similar samples was increased, while the distance between dissimilar samples was unaffected. The filter protocol did not distort overall community compositions.

The AmpliconDuo filter was not an effective means for the removal of chimeras, especially not for the astonishingly frequent chimeras with relatively high read numbers in prokaryotic data. The latter fact points to a high degree of non-randomness in chimera generation. Thus, if chimera removal is required, application of other specialized methods is necessary.

We have demonstrated with a simple model based on the Poisson distribution, that the AmpliconDuo filter in most cases removes sequences with read numbers of up to ten. This does not mean that working with a non-split sample and removing all sequences with up to ten reads has the same effect. First, this would remove many more rare true positive sequences that occur in both branches. Second, there are some instances where the filter removes sequences with much higher read numbers that occur in only one branch, a pattern expected e.g. from artifacts that were formed in early PCR cycles.

As HTSeq has made great strides in terms of cost efficiency and sequencing depth, and as there is still no end to this development, the argument that split samples sacrifice too much of the sequencing depth becomes less and less relevant. On the contrary, as we have demonstrated in this work, important information to assess and improve the quality of the data, in particular for rare OTUs where sequencing depth is critical, becomes available with the split sample approach and is difficult to obtain in other ways.

## Supporting Information

S1 Fig Discordance plot showing significant deviations of prokaryote read numbers between split samples. For legend see Fig 3.
(TIF)

S2 Fig Effect of AmpliconDuo filter on spectrum of read numbers for prokaryotic data. Axes as in Fig 4.
(TIF)

S3 Fig Relatedness of eukaryote sequences discarded by AmpliconDuo filter to sequences passing the filter. The two columns A, B are the experimental branches, rows are sampling sites. Horizontal axes are read numbers of discarded sequences. Vertical axes are percentages of discarded sequences in a Levenshtein distance of 1 to 4 editing operations to passing sequences. Numbers above bars are absolute numbers of sequences. Example: first bar in branch A of bog soil sample contains 733 sequences (=100%) that were removed by AmpliconDuo filter. 62% of these 733 sequences have a Levenshtein distance of *L* = 1 to retained sequences (brown bar), 78% have a distance of *L* ≤ 2 (upper edge of red bar), for 85%: *L* ≤ 3 (upper edge orange bar), for 87%: *L* ≤ 4 (upper edge yellow bar). The remaining 100%-87% = 13% have *L* > 4 to sequences that pass AmpliconDuo filter.
(TIF)

S4 Fig Relatedness of prokaryote sequences discarded by AmpliconDuo filter to sequences passing the filter. Legend as in S3 Fig.
(TIF)

S5 Fig Effect of UCHIME and AmpliconDuo filter on prokaryotic data. For the two prokaryotic samples Pro1 and Pro3 (left and right column), the figure compares numbers of sequences discarded by the AmpliconDuo filter (top row), by removal of all sequences recognized by UCHIME *de novo* as chimeras (middle row), and by the combination of both (bottom row). Numbers on top of the bars are absolute frequency counts of sequences with the numbers of reads indicated on the horizontal axis. AmpliconDuo filter has a perceivable effect only on low abundance chimeras. For higher abundance chimeras (again above about 10 reads), the middle and bottom rows are virtually the same.
(TIF)

S1 Table Poly-N region and sample identifiers for all eukaryotic samples and respective experimental branches A and B. (PDF)

S2 Table Poly-N region and identifiers for all prokaryotic samples and respective experimental branches A and B. (PDF)

S3 Table Percentages of sequences and reads removed by application of the AmpliconDuo filter from both branches A and B of each split sample. (PDF)

S4 Table Sequence richness in all eukaryotic samples before and after application of AmpliconDuo filter. Taxa not addressed in the analysis (Bacteria, Metazoa or Embryophyta) were discarded (see also section Community comparison in Materials and Methods).
(PDF)
