# Supplementary figures and images for: AmpliconDuo: A Split-Sample Filtering Protocol for High-Throughput Amplicon Sequencing of Microbial Communities
Source: PLoS One. 2015 Nov 2;10(11):e0141590. doi: 10.1371/journal.pone.0141590 (PMC4629888; doi:10.1371/journal.pone.0141590)

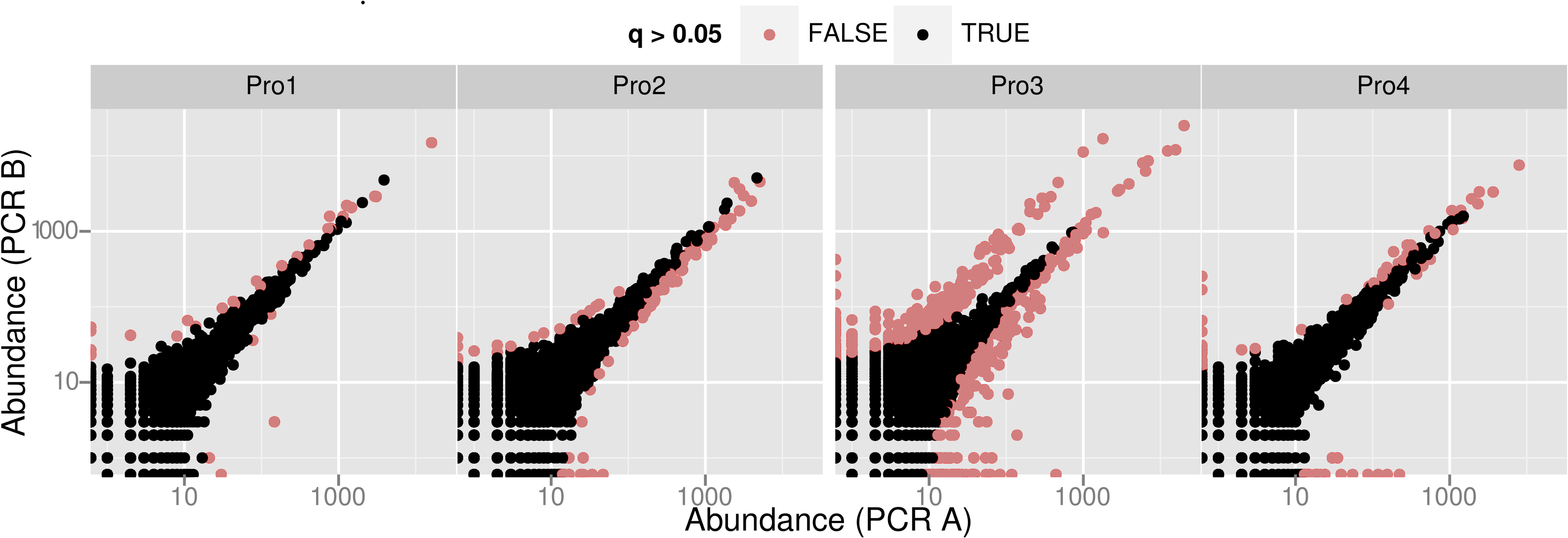

Supplement: S1 Fig — For legend see Fig 3. (TIF) [file pone.0141590.s001.tif]

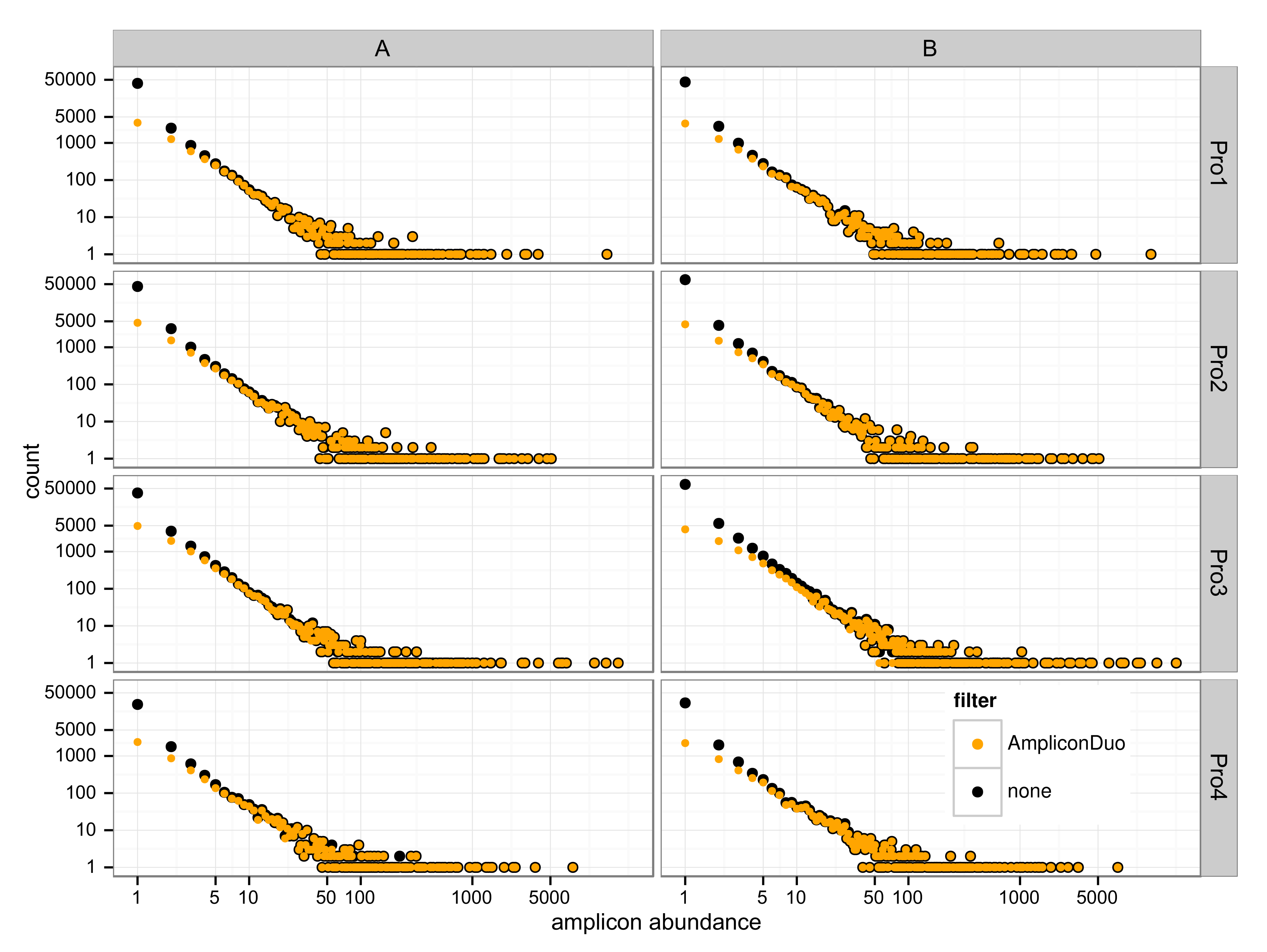

Supplement: S2 Fig — Axes as in Fig 4. (TIF) [file pone.0141590.s002.tif]

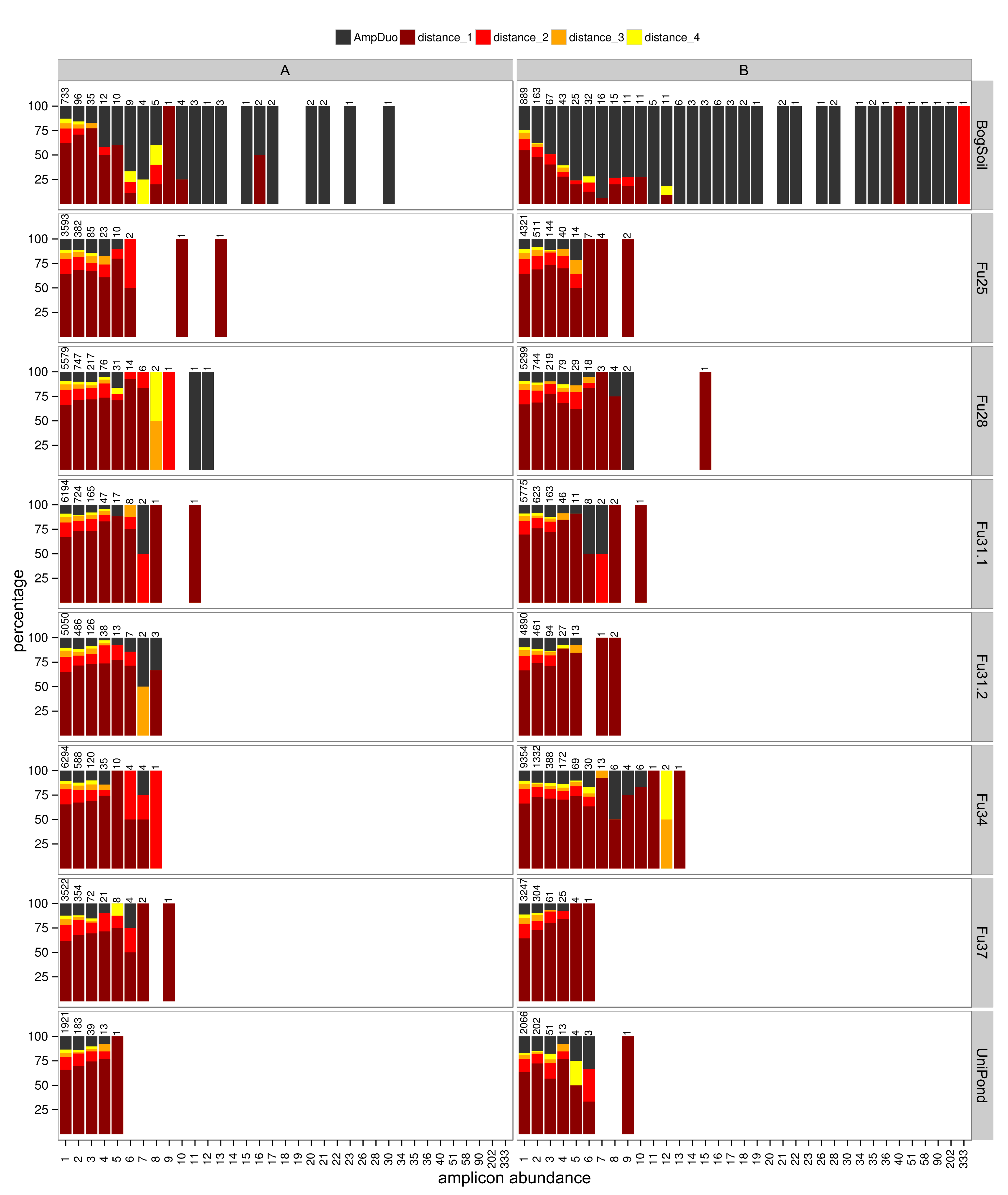

Supplement: S3 Fig — The two columns A, B are the experimental branches, rows are sampling sites. Horizontal axes are read numbers of discarded sequences. Vertical axes are percentages of discarded sequences in a Levenshtein distance of 1 to 4 editing operations to passing sequences. Numbers above bars are absolute numbers of sequences. Example: first bar in branch A of bog soil sample contains 733 sequences (=100%) that were removed by AmpliconDuo filter. 62% of these 733 sequences have a Levenshtein distance of L = 1 to retained sequences (brown bar), 78% have a distance of L ≤ 2 (upper edge of red bar), for 85%: L ≤ 3 (upper edge orange bar), for 87%: L ≤ 4 (upper edge yellow bar). The remaining 100%-87% = 13% have L > 4 to sequences that pass AmpliconDuo filter. (TIF) [file pone.0141590.s003.tif]

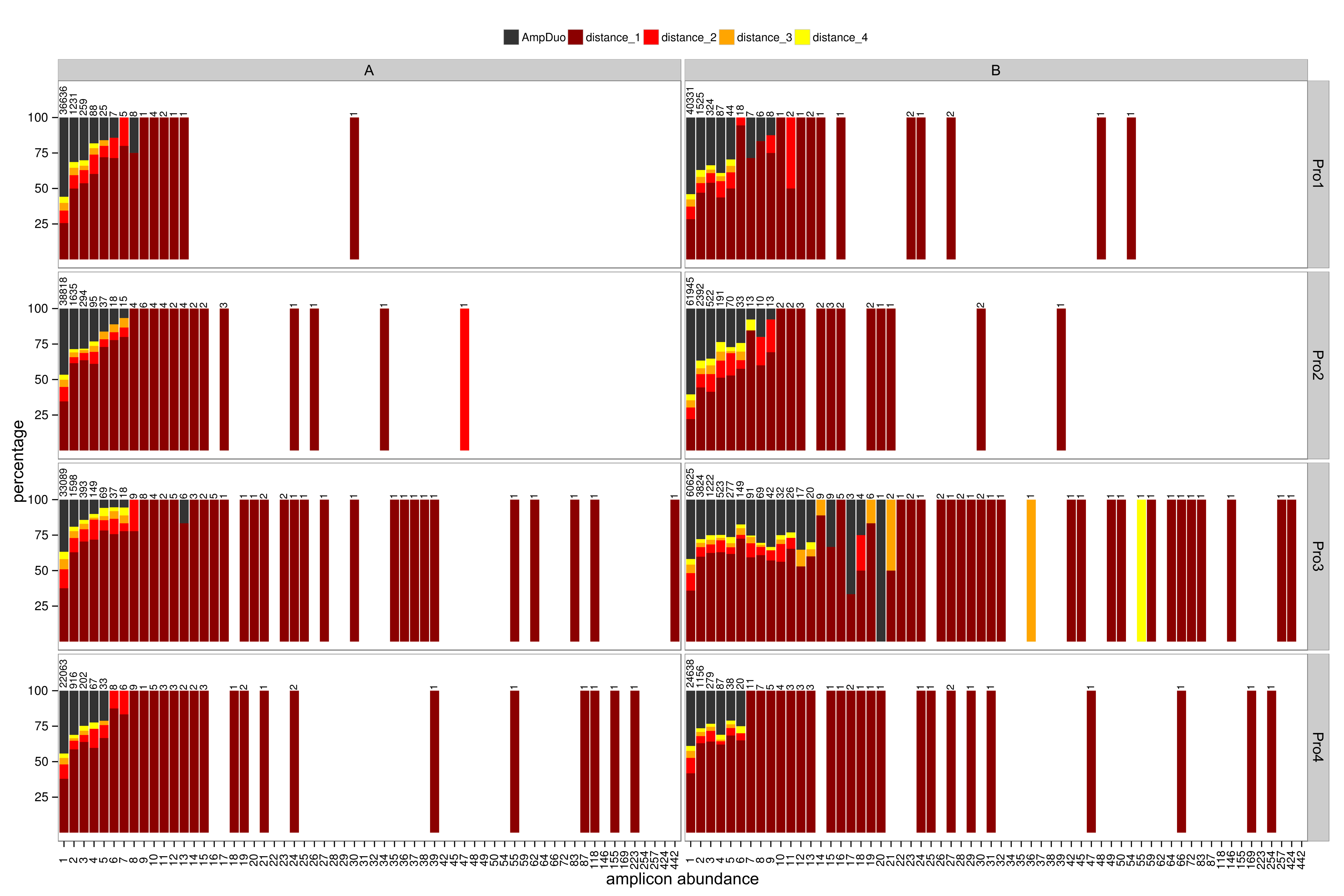

Supplement: S4 Fig — Legend as in S3 Fig. (TIF) [file pone.0141590.s004.tif]

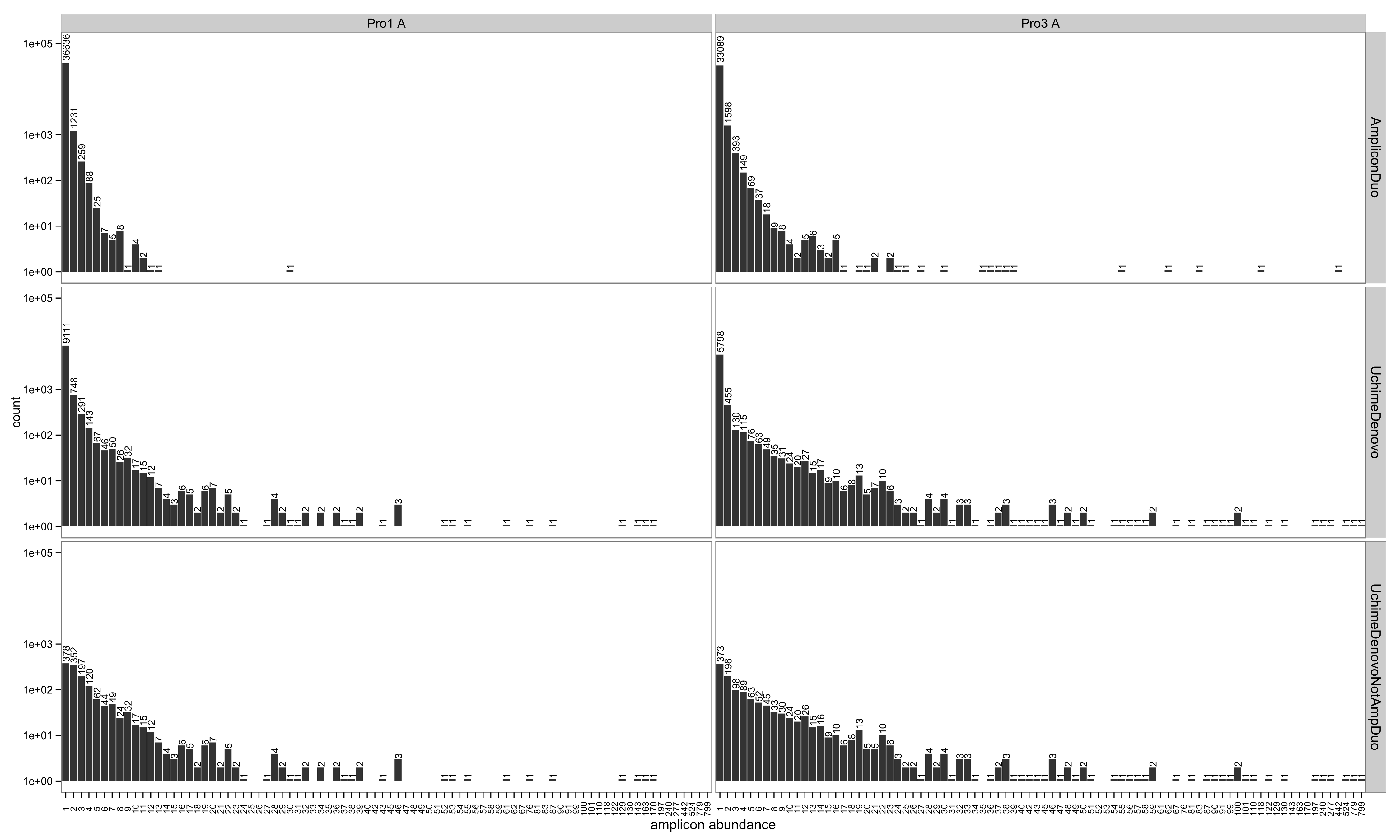

Supplement: S5 Fig — For the two prokaryotic samples Pro1 and Pro3 (left and right column), the figure compares numbers of sequences discarded by the AmpliconDuo filter (top row), by removal of all sequences recognized by UCHIME de novo as chimeras (middle row), and by the combination of both (bottom row). Numbers on top of the bars are absolute frequency counts of sequences with the numbers of reads indicated on the horizontal axis. AmpliconDuo filter has a perceivable effect only on low abundance chimeras. For higher abundance chimeras (again above about 10 reads), the middle and bottom rows are virtually the same. (TIF) [file pone.0141590.s005.tif]
