# Supplementary material for: AmpliconDuo: A Split-Sample Filtering Protocol for High-Throughput Amplicon Sequencing of Microbial Communities
Source: PLoS One. 2015 Nov 2;10(11):e0141590. doi: 10.1371/journal.pone.0141590 (PMC4629888; doi:10.1371/journal.pone.0141590)
Supplement: S1 Table — (PDF) [file pone.0141590.s006.pdf]

**ID**	**Sample**	**Poly-N region**	**MID**
SSU 1A	FU31.1	NNNNN	ACAGAGCA
SSU 1B	FU31.1	NNNNN	AATGCCAA
SSU 1C	FU31.2	NNNNN	AGCATTGA
SSU 1D	FU31.2	NNNNN	ATGAGAGA
SSU 2A	FU25	NNNNN	TGACTGATT
SSU 2B	FU25	NNNNN	TTTCGCATG
SSU 3A	FU28	NNNNN	TACACTGTC
SSU 3B	FU28	NNNNN	TCGTAAGTA
SSU 4A	FU34	NNNNN	CAATCGTCAT
SSU 4B	FU34	NNNNN	CGTATCACGT
SSU 5A	FU37	NNNNN	CCCTATACTT
SSU 5B	FU37	NNNNN	CTGAGATCCT
SSU 6A	Uni Pond	NNNNN	GGATTGTGGTA
SSU 6B	Uni Pond	NNNNN	GTTAGCTGCAA
SSU 7A	Bog Soil	NNNNN	GCGTAACGTAG
SSU 7B	Bog Soil	NNNNN	GAGTCAAGATG
