# Supplementary material for: AmpliconDuo: A Split-Sample Filtering Protocol for High-Throughput Amplicon Sequencing of Microbial Communities
Source: PLoS One. 2015 Nov 2;10(11):e0141590. doi: 10.1371/journal.pone.0141590 (PMC4629888; doi:10.1371/journal.pone.0141590)
Supplement: S2 Table — (PDF) [file pone.0141590.s007.pdf]

ID	Sample	Poly-N fwd	MID	Poly-N rev
KO1 A	Pro1	NNNNN	TCTGAAACGCAA	NNNN
KO1 B	Pro1	NNNNNN	TACCATTTGCTC	NNNN
KO3 A	Pro2	NNNNN	GTCACACTTGCG	NNNNNN
KO3 B	Pro2	NNNNNN	GATGCCTCTAAC	NNNNNN
NO3.2 A	Pro3	NNN	GCGCCGCATATA	NNNNN
NO3.2 B	Pro3	NNNN	ACATGCAGCCAA	NNNNN
NP3 A	Pro4	NNN	CTCCTCCTAGTG	NNN
NP3 B	Pro4	NNNN	TTCAAACTGGCG	NNN
