# Supplementary material for: AmpliconDuo: A Split-Sample Filtering Protocol for High-Throughput Amplicon Sequencing of Microbial Communities
Source: PLoS One. 2015 Nov 2;10(11):e0141590. doi: 10.1371/journal.pone.0141590 (PMC4629888; doi:10.1371/journal.pone.0141590)
Supplement: S3 Table — (PDF) [file pone.0141590.s008.pdf]

sample	ampliconsA	readsA	ampliconsB	readsB
BogSoil	58.21	3.68	66.68	8.34
FU25	63.48	6.25	68.15	6.48
FU28	64.52	5.09	63.55	4.55
Fu31.2	63.98	5.16	63.00	4.99
Fu31.1	63.69	4.89	61.90	4.34
FU34	54.13	3.23	65.55	3.70
FU37	62.99	2.30	60.87	2.06
UniPond	55.29	1.56	57.43	1.73
Pro1	84.41	32.26	85.70	32.04
Pro2	82.53	29.06	88.27	40.14
Pro3	77.36	19.84	86.60	24.98
Pro4	82.81	27.43	84.43	26.42
