# Supplementary material for: AmpliconDuo: A Split-Sample Filtering Protocol for High-Throughput Amplicon Sequencing of Microbial Communities
Source: PLoS One. 2015 Nov 2;10(11):e0141590. doi: 10.1371/journal.pone.0141590 (PMC4629888; doi:10.1371/journal.pone.0141590)
Supplement: S4 Table — Taxa not addressed in the analysis (Bacteria, Metazoa or Embryophyta) were discarded (see also section Community comparison in Materials and Methods). (PDF) [file pone.0141590.s009.pdf]

	#reads AmpDuo	#reads no Filter	#OTUs AmpDuo	#OTUs no Filter
BogSoil A	38699	40053	510	1255
BogSoil B	40317	43763	510	1548
FU25 A	69871	74517	2303	6281
FU25 B	85725	91644	2303	7194
FU28 A	150619	158673	3551	9958
FU28 B	164750	172597	3551	9722
FU31.1 A	143196	151078	3757	10406
FU31.1 B	150524	157805	3757	9915
FU31.2 A	106591	112873	2955	8313
FU31.2 B	103562	109385	2955	8038
FU34 A	228694	236391	5671	12373
FU34 B	358170	371874	5671	16276
FU37 A	57442	61633	1915	5535
FU37 B	56337	60130	1915	5230
UniPond A	150018	152327	1662	3690
UniPond B	148975	151566	1662	3886
